# Defeating Paediatric Tuberculous Meningitis: Applying the WHO “Defeating Meningitis by 2030: Global Roadmap”

**DOI:** 10.3390/microorganisms9040857

**Published:** 2021-04-16

**Authors:** Robindra Basu Roy, Sabrina Bakeera-Kitaka, Chishala Chabala, Diana M Gibb, Julie Huynh, Hilda Mujuru, Naveen Sankhyan, James A Seddon, Suvasini Sharma, Varinder Singh, Eric Wobudeya, Suzanne T Anderson

**Affiliations:** 1 Clinical Research Department, Faculty of Infectious & Tropical Diseases, London School of Hygiene & Tropical Medicine, Keppel Street, London WC1E 7HT, UK; 2 MRC Clinical Trials Unit at UCL, 90 High Holborn, Holborn, London WC1V 6LJ, UK; diana.gibb@ucl.ac.uk (D.M.G.); suzanne.anderson@ucl.ac.uk (S.T.A.); 3 MUJHU Research Collaboration, Kampala, Uganda; sabrinakitaka@yahoo.co.uk (S.B.-K.); ewobudeya@mujhu.org (E.W.); 4 School of Medicine & University Teaching Hospital (UTH), University of Zambia, Lusaka, Zambia; cchabala@gmail.com; 5 Oxford University Clinical Research Unit, Centre for Tropical Medicine, Hospital for Tropical Diseases, 764 Vo Van Kiet, District 5, Ho Chi Minh City, Vietnam; jhuynh@oucru.org; 6 Centre for Tropical Medicine and Global Health, Nuffield Department of Medicine, Oxford University, Oxford OX3 7LG, UK; 7 University of Zimbabwe Clinical Research Centre, Harare, Zimbabwe; hmujuru@mweb.co.zw; 8 Post Graduate Institute of Education and Medical Research (PGI), Chandigarh 160017, India; drnsankhyan@yahoo.co.in; 9 Department of Infectious Diseases, Imperial College London, Norfolk Place, London W2 1PG, UK; james.seddon@imperial.ac.uk; 10 Desmond Tutu TB Centre, Department of Paediatrics and Child Health, Stellenbosch University, Cape Town 8000, South Africa; 11 Department of Pediatrics, Lady Hardinge Medical College and Assoc Kalawati Saran Children’s Hospital (Hospital-LHH), New Delhi 110001, India; sharma.suvasini@gmail.com (S.S.); 4vsingh@gmail.com (V.S.)

**Keywords:** tuberculosis, tuberculous meningitis, TBM, children

## Abstract

Children affected by tuberculous meningitis (TBM), as well as their families, have needs that lie at the intersections between the tuberculosis and meningitis clinical, research, and policy spheres. There is therefore a substantial risk that these needs are not fully met by either programme. In this narrative review article, we use the World Health Organization (WHO) “Defeating Meningitis by 2030: global roadmap” as a starting point to consider key goals and activities to specifically defeat TBM in children. We apply the five pillars outlined in the roadmap to describe how this approach can be adapted to serve children affected by TBM. The pillars are (i) prevention; (ii) diagnosis and treatment; (iii) surveillance; (iv) support and care for people affected by meningitis; and (v) advocacy and engagement. We conclude by calling for greater integration between meningitis and TB programmes at WHO and at national levels.

## 1. Introduction

Tuberculous meningitis (TBM) is a devastating childhood disease, with one in five affected children dying and only one in three surviving without disability [1]. Depending upon tuberculosis prevalence, the age range being studied, and whether it is a population or hospital-based setting, TBM accounts from 1 to 10% of childhood TB cases, although there is considerable uncertainty around these estimates, given the challenges of making a microbiological diagnosis, the difficulties in discriminating TBM from other meningitides on clinical grounds, and underreporting [2,3]. The forthcoming publication of the first global road map on defeating meningitis from the World Health Organization (WHO) provides an opportune moment to highlight the specific challenges and goals needed to defeat TBM [4]. Issues affecting children with TBM and their families lie at the intersection of the tuberculosis (TB) and meningitis clinical, research, and policy spheres and, hence, there is an accompanying risk that the specific needs of this vulnerable group are not fully addressed (Table 1).

In this article we therefore apply the framework outlined in the October 2020 draft “defeating meningitis” road map to TBM. As a team of paediatricians working on the SURE trial, the largest ever randomised controlled treatment trial in paediatric TBM, we focus specifically upon TBM in children [7]. The “defeating meningitis” roadmap explicitly places its emphasis on the four main causes of acute bacterial meningitis (meningococcus, pneumococcus, *Haemophilus influenzae,* and group B streptococcus), although it also highlights that many of the goals are applicable to reducing the burden of disease from all causes of meningitis (Table 1) [4]. The “defeating meningitis” roadmap sits within a network of interconnected issues related to TB including universal health coverage, antimicrobial resistance and inclusion of people with disabilities [4]. Its vision is “Towards a world free of meningitis,” although this is qualified, as meningitis is heterogenous and not amenable to elimination or eradication. Three overarching visionary goals are proposed: (i) elimination of bacterial meningitis epidemics; (ii) reduction of cases and deaths from vaccine-preventable bacterial meningitis; (iii) reduction of disability and improved quality of life after meningitis of any cause [4]. From the outset, the limitations of applying the roadmap to paediatric TBM are clear, as although it is, to some extent, vaccine-preventable and a significant cause of disability, it is not a disease that occurs in epidemics [1,8].

We consider the five pillars of (i) prevention; (ii) diagnosis and treatment; (iii) disease surveillance; (iv) support and care for people affected by meningitis; and (v) advocacy and engagement, to outline how this approach can be adapted to serve children affected by TBM. We conclude by calling for greater integration of the approaches to eliminate TB and defeat meningitis, especially in light of the challenges and opportunities presented to healthcare systems around the world tackling the COVID-19 pandemic.

## 2. Pillar 1: Prevention

Prevention of paediatric TBM is closely related to the prevention of TB infection and thereby TB disease, and severe forms of disease such as TBM. Strategies to control TBM should focus on TB prevention with a special focus on vulnerable groups. In regions with high TB prevalence, children under 5 years are most commonly affected with TBM [2,9]. Others at risk of TBM include those living with HIV and other immunocompromised children. The WHO lists the three primary strategies for TB prevention, including (i) TB preventive therapy (TPT); (ii) prevention of transmission through infection control; and (iii) BCG vaccination [5]. It is recommended to use TPT to prevent TB infection from progressing to TB disease. This is particularly important in immunocompromised hosts and young children in whom the risk of severe forms of TB, including TBM, is high. The WHO recommends TPT for three high-risk groups: household contacts (especially children under five years of age), people living with HIV, and other clinical risk groups [10]. This latter group, which includes those initiating anti-TNF treatment or transplantation, prisoners, health workers, homeless people, and people who use drugs, represents a relatively small proportion of children at risk in high TB prevalence areas.

In March 2020, WHO updated recommendations on TPT [10]. Alongside established regimes, such as 6 or 9 months of daily isoniazid, 3 months of weekly rifapentine plus isoniazid, and 3 months of daily isoniazid and rifampicin, new recommendations included:

- A 1-month regimen of daily rifapentine and isoniazid (1HP);
- A 4-month regimen of rifampicin (4R);
- Isoniazid preventive treatment in pregnancy; and
- Advice on the use of rifapentine and dolutegravir for people living with HIV.

The inclusion of rifampicin and rifapentine in regimens for TB prevention allows shorter treatment and higher completion rates. There is ongoing research to improve uptake and scale-up TPT, such as the Unitaid-funded: IMPAACT 4TB and CaP TB studies [5]. Despite clear guidance, the numbers of household contacts, including children, provided with TPT globally, remain dismally low at 20–30% [5,10,11]. It is therefore not surprising that, in a case series of children with TBM, TPT was not provided to any of the children who had known exposure to an adult TB case [12].

TB infection, prevention and control is part of the End TB strategy. Particular areas of concern for preventing TB transmission through infection control include:

- Newborn care settings. There are many documented outbreaks of TB among neonates with the source case usually being a mother or a member of staff with potential to infect many babies in the neonatal care unit. Neonates are particularly vulnerable for acute onset or development of disseminated severe disease [13,14].
- Health facilities that provide care for adults, older children, and adolescents with TB, who are often infectious.
- Antenatal care settings.
- HIV clinics, including Preventing Mother to Child Transmission (PMTCT) settings.
- Facilities that care for children with severe malnutrition.
- Other congregate settings, including childcare facilities, orphanages, prisons, and schools. For older children, this includes boarding schools. School-aged children with sputum smear-positive TB should be kept from attending school until it is considered that there is a very low risk of transmission. This is usually for 2 weeks in the case of drug-susceptible TB.
- Children in displaced and mobile populations, including migrant labour camps, informal and crowded refugee camps, and temporary shelters.

In 2019, WHO released new guidance, and recommended administrative, environmental, and personal protection measures to limit the spread of TB [15]. The COVID-19 pandemic has threatened to derail the global progress in TB control. Alarmingly, it has been estimated that the COVID-19 pandemic could cause an additional 6.3 million TB cases globally between 2020 and 2025 [16,17]. However, there are some positives too. The expertise and experience in rapid testing and contact tracing for COVID-19 could be leveraged for TB case tracing and testing. Additionally, digital communication strategies developed during COVID-19 could be expanded for remote care, follow-up, and support of TB patients [18,19]. Lastly, the enhanced awareness generated during the COVID-19 pandemic could help emphasise basic infection prevention strategies in healthcare settings, such as the use of personal protective equipment (PPE), cough etiquette, and respiratory isolation [5].

The third key strategy is neonatal BCG vaccination. This has been shown to protect against TBM and disseminated TB in children with protection lasting up to the age of 10 years [8,20,21]. Data on protection beyond 15 years are limited; however, a small number of trials and observational studies suggest that BCG vaccination may protect for longer. Importantly, there are limitations to the potential for BCG to further decrease incidence of TBM in childhood, as the majority of children with TBM have been vaccinated with BCG. An investigational TB vaccine candidate (M72/AS01E) was evaluated in adults infected with *Mycobacterium tuberculosis* (*M. tuberculosis)*. The vaccine efficacy at month 36 was 49.7% (90% confidence interval [CI], 12.1 to 71.2; 95% CI, 2.1 to 74.2) in prevention of TB disease [22]. It remains to be seen if this vaccine will prove to have the effectiveness needed for more widespread use (Table 2).

## 3. Pillar 2: Diagnosis and Treatment

Early and accurate diagnosis of TBM remains challenging. Diagnostic and treatment delay are the most important predictors of mortality and disability [23]. The challenges are numerous: (1) non-specific symptoms leading to misdiagnosis; (2) inadequately sensitive diagnostic tools to confirm TBM; (3) suboptimal sample collection for laboratory processing; and (4) lack of standardised region-specific training of healthcare staff to identify TBM and initiate appropriate anti-TB therapy.

Mycobacterial cultures are considered the “gold standard” diagnostic test for TBM. However they take 2–6 weeks to yield a result and require specialised laboratory facilities and experience not available at all healthcare levels. The rapid molecular test, Xpert MTB/RIF, has emerged as an important diagnostic test for all forms of TB, such that WHO have recommended it as the initial TB diagnostic test, replacing the acid-fast bacilli smear [24]. Next-generation Xpert MTB/RIFUltra (Xpert Ultra) has a sensitivity of 44–77% and specificity approaching 100% in the cerebrospinal fluid (CSF) of adults with TBM [25,26,27]. Xpert MTB/RIF offers value for clinical decision-making as it allows early initiation of anti-TB therapy and information on the presence/absence of rifampicin resistance and, hence, whether treatment for multidrug-resistant (MDR) TBM is indicated. However, the test requires an expensive platform, access to electricity, and costly consumables. Xpert cartridges costs USD $10 in countries that are allowed concessional pricing; however, for high TB burden countries, this equates to a substantial expenditure [28]. Although the next generation Xpert Ultra has slightly higher sensitivity than Xpert MTB/RIF it does not have a sufficiently high negative predictive value to exclude TBM when the result is negative. Microbiological confirmation of MDR-TB in children is uncommon. In the absence of a microbiological diagnosis, MDR-TBM requires careful history-taking including recent exposure to an infectious MDR-TB source case or someone who failed TB treatment or died from suspected MDR-TB.

Xpert MTB/RIF and Xpert Ultra should not be used as the sole diagnostic test in TBM and every attempt should be made to obtain samples from elsewhere such as sputum, gastric aspirates, lymph node aspirates, biopsies, to support the diagnosis. Recent rapid diagnostic tests, such as TB-LAMP (loop-mediated isothermal amplification), which amplifies MTB DNA and is implementable at peripheral health centre level, and urinary TB FujiLAM, which detects *M. tuberculosis* lipoarabinomannan antigen, remove the need for advanced laboratory expertise [29,30,31]. However, information on their role in TBM diagnosis, diagnostic performance in the paediatric population, and impact on mortality have yet to be clearly elucidated. In the absence of an optimal and accessible diagnostic test, diagnosis of TBM still relies on clinical symptoms and signs, CSF parameters, and radiology (chest radiographs at the minimum and neuroimaging where available), and, where available, bacterial, viral and fungal microbiological tests to distinguish TBM from other infective causes of meningitis. A clinical decision tool (i.e., CHILD TB LP- altered Consciousness, caregiver HIV-infected, Illness length, Lethargy, focal neurological Deficit, failure to Thrive, Blood/serum sodium, CSF Lymphocytes, CSF Protein) developed to facilitate early diagnosis of childhood TBM has been shown to accurately classify microbiologically confirmed TBM (sensitivity 100%, specificity 90%) [32]. However, this algorithm requires prospective evaluation as a rapid diagnostic tool in children with CNS infections in numerous geographical settings with varying TB burden and risk factors.

Bacteriologic confirmation of meningitis caused by *M. tuberculosis* relies heavily on obtaining adequate volumes of CSF (at least 6 mL for mycobacterial testing and additional volume for tests to exclude other causes of meningitis, e.g., pyogenic bacterial meningitis, *Cryptococcus* sp., and viral meningoencephalitis) [33]. As TBM is paucibacillary in nature, maximising the number of mycobacteria in a sample will increase the probability of a positive result. This is achieved by obtaining large volumes of CSF with subsequent centrifugation (3000× *g* for 15 min) to concentrate *M. tuberculosis* into pellet form [34]. Increased focus on the importance of CSF collection and processing should be incorporated into TB guidelines, and in training of healthcare and laboratory staff (Table 3). Even in the absence of positive TB microbiology, CSF taken for microscopy and biochemistry is important for TBM diagnosis. This highlights the pivotal role of frontline clinicians in obtaining informed consent for the lumbar puncture as part of the routine diagnostic approach. Parental refusal to perform lumbar punctures in children with suspected central nervous system infection is common in low-middle income (LMIC) settings; a cross-sectional study of 215 families of Pakistani children who had indications for lumbar puncture showed that 33% refused [35]. Common reasons for refusal were lack of knowledge about the risks of the procedure (30%) and fear of paralysis of lower limbs (49%). High levels of illiteracy, stigma associated with the procedure and potential differences of opinion amongst the extended family impact on LP consent in many LMIC settings. Training of healthcare staff on how to counsel families in these settings, and guidance on the process of informed consent with real-world scenarios may reduce the frequency of parental refusal for LP, given its importance to securing a diagnosis of TBM. Current research is focussed on detecting novel blood, CSF, and urine biomarkers (transcriptome, proteome, and metabolome) to diagnose TBM in children [36,37,38,39]. Such approaches and new biomarkers of cerebral injury in CSF offer much promise for future diagnosis of TBM in children [40].

The treatment of TBM is urgent to limit disability and death, especially in a condition with delayed presentation and diagnosis. The WHO regimen for drug susceptible TBM is two months of HRZE ((INH: 10 mg/kg), rifampicin (RMP: 15 mg/kg), pyrazinamide (PZA: 35 mg/kg), and ethambutol (EMB: 20 mg/kg) (HRZE)) intensive phase followed by a continuation phase of ten months of HR treatment, which is 6 months longer than the treatment for pulmonary TB. The 2 HRZE used in the crucial initial 2 months of TB treatment is recognised to be suboptimal in penetrating the blood–brain barrier and new regimens are being put into clinical trials [7,41,42]. The pathophysiology of TBM includes severe inflammation in the brain with potential for raised intracranial pressure, hydrocephalus, ischaemia, and infarction [43]. Adjuvant treatment with steroids in the initial 6–8 weeks has been shown in a Cochrane systematic review to improve mortality, but to have little effect on disabling sequelae [44]. However, the benefits of immunomodulatory treatment may depend on genetically regulated levels of inflammation [45]. More recently, data from an adult treatment trial of TBM suggests that addition of adjunctive aspirin may improve clinical outcomes in certain patient subgroups at high dose and this is under evaluation in an ongoing paediatric clinical trial [7,46].

The optimal dosing, choice, route and duration of antitubercular agents in TBM and the context of an inflamed blood brain barrier remains the subject of debate. In Cape Town, South Africa, children with TBM are treated with a shorter, higher dose 6 month regimen: INH: 20 mg/kg, RMP: 20 mg/kg, PZA: 40 mg/kg and ethionamide: 20 mg/kg for 6 months [47]. Although excellent outcomes have been reported with this “Cape Town” regimen it has never been subjected to a randomised controlled trial and it is not clear whether the apparent improvements are due to the regimen or high quality supportive care [47]. Given the potential to inform global TB policy with improved CSF penetration, halving the duration of treatment and hence improving concordance, the recently commenced SURE trial will compare the 12-month WHO regimen with a modified 6-month intensified regimen (RMP 30 mg/kg; INH 20 mg/kg; ethionamide replaced with levofloxacin, which penetrates CSF well) in children under 15 years [7]. The impact of adjunctive high dose aspirin vs placebo on severe disability will also be evaluated.

Successful treatment of MDR-TBM in children presents further challenges. MDR-TB meningitis is associated with high mortality owing to delayed diagnosis of drug resistance, the absence of a standardised approach to the management and poor CSF penetration of many MDR-TB drugs [48,49]. Whilst it is recommended that treatment regimens for MDR-TB should include at least 4–5 effective drugs, and careful consideration of drugs that penetrate the CSF well such as fluoroquinolones and linezolid, there is a lack of evidence to inform best antibiotic combination, duration and doses [50]. In light of the increasing threat of MDR-TB, pre-clinical studies evaluating CSF-brain penetrating properties of new TB drugs and clinical trials assessing optimal drug regimens to improve outcome are urgently needed (Table 3). 

## 4. Pillar 3: Disease Surveillance

Systems for TB surveillance are some of the oldest in the world, having begun more than two centuries ago with the recording of TB mortality in England and Wales [51]. As defined by the WHO, the primary aim of global TB surveillance is to assess the progress of TB control activities in the context of the End TB strategy [5]. WHO collates all the annual surveillance data provided by countries and then generates global estimates. These are adjusted by correction factors to account for underreporting, over- and under-diagnosis [5]. Globally, an estimated 10 million (range, 8.9–11.0 million) people fell ill with TB in 2019 [5]. Extrapulmonary TB represented 16% of the 7.1 million incident cases that were notified in 2019 [5].

The backbone of TB surveillance is case notification, which is statutory in many countries. The most accepted international case definitions for notification are the 2013 WHO definitions, last updated in January 2020, which incorporate: bacteriological status, classification of disease as pulmonary and extra-pulmonary, history of previous TB treatment, HIV status, and drug resistance [52]. Surveillance is incorporated within the national TB control programs in most LMIC as a monitoring and evaluation tool. The focus of surveillance at present are the infectious pulmonary cases for which more data are provided, while all extra-pulmonary forms are reported collectively. For example, the Global TB report provides no further details about extra-pulmonary TB including TBM [5].

Use of electronic registers for TB cases allows the capture of more detailed information such as a breakdown of the extra-pulmonary sites of disease but these are not part of formal reporting at national and global level. The National TB Elimination Program of India kindly shared data on CNS TB from their national e-Register for this publication. They reported that CNS TB among under 18 years olds contributed to 1.06%, 1.27%, and 1.66% of the total extra-pulmonary TB cases in the years 2018, 2019 and 2020, respectively (personal communication to Varinder Singh).

Amongst the other challenges that compromise detailed surveillance for TBM is the lack of a clear case definition. As paediatric TBM is a paucibacillary disease, many infectious and non-infectious neurological diseases may be misdiagnosed as TBM, such as partially treated bacterial meningitis, viral encephalitis, autoimmune encephalitis, and subacute onset neurodegenerative diseases. This is compounded by a paucity of neuroimaging facilities or facilities for molecular TB diagnostics in low resource settings. TBM, despite its own specific diagnostic and therapeutic challenges, is neglected in most TB programmatic guidelines, which typically only address the duration of treatment and adjunctive corticosteroids [53,54]. Both under- and over-reporting of TBM, as a result of misdiagnosis, is also likely in low-resource settings.

As TBM diagnosis requires some key laboratory facilities and clinical expertise, it is often centralised to large hospitals. Thus, TBM surveillance requires data from hospitals, both in the public and private sector, and also from laboratories where microbiological testing is performed. Even in public hospitals, reporting has many challenges. In a study from China, 25% of cases that were documented in the hospital records were not reported to the public health authorities [55,56]. Factors cited that also apply in other settings were unqualified and overworked health personnel, poor supervision and accountability at local and national levels, and a complicated incohesive health information management system [55,56]. The situation is complex in the case of private practitioners as the system requires them to provide patient details from outside the state health system. Limited practitioner time is an important barrier to TB notification and therefore user-friendly interventions, such as mobile based notification, have potential to improve notification, although are constrained by technology and internet access [57]. In a pilot study of the use of a mobile interface voice-based TB notification system, only 6% of private practitioners were found to use it [57].

Although TB surveillance is one of the most well-established and prevalent surveillance systems across the globe, there is a need for a well-defined strategy for TBM surveillance, which is poorly quantified at present. The data thus collected will be helpful to understand the disease, its trends and inform appropriate action (Table 4).

## 5. Pillar 4: Support and Care for People Affected by Meningitis

For many childhood survivors of TBM, the majority of whom live in LMICs, severe illness results in significant neurodevelopmental sequelae. A meta-analysis on treatment outcomes in childhood TBM demonstrated neurological sequelae in 54% of survivors [1]. However, data on the physical, cognitive, and behavioural sequelae of TBM, which have lasting socioeconomic implications for patients and their families, are limited and rarely include long-term follow-up. Children living with disabilities in LMICs are likely to experience poorer health and quality of life, reduced school participation and high rates of poverty when compared with their non-disabled peers [58,59], yet this has not been evaluated in TBM survivors.

Common impairments documented post TBM are in cognition, learning, emotion, and behaviour, all potentially affecting educational attainment and future employment. Poor neurodevelopmental outcome is associated with younger age, delayed presentation and treatment initiation, clinical severity and hydrocephalus, highlighting the need for increasing awareness of TBM and better clinical and diagnostic tools for timely initiation of treatment and management of sequelae [9,60,61].

The United Nations Sustainable Development Goal (SDG) 4, Pillar 4 of the Roadmap to Defeating Meningitis by 2030, and United Nations Convention on the Rights of the Child together highlight the need for timely identification and management of sequelae, together with reliable, valid measures to evaluate preventive and interventional efforts as well as improved access to appropriate support and care services [4,62,63]. Achieving these goals, considered standard of care in most high-income countries, will be challenging for several reasons.

First, there is a paucity of robust and standardised neurodevelopmental assessment tools (NDATs) developed for, and with normal reference populations, across different geographical and cultural settings. To fully understand the burden of impairment caused by TBM will, therefore, require appropriately adapted, as well as new, locally developed NDATs to detect both early developmental and later, emergent speech, behavioural and cognitive difficulties together with adaptive function [64]. A number of NDATs that have either been adapted for use in or designed for different geographical and cultural settings are now being used [65]. However, these often require a high level of skill and training for healthcare professionals and are time-consuming. Screening tools to detect childhood disability and suitable for use by a broader range of healthcare workers are evolving. For example, the WHO Disability Assessment Schedule (DAS) 2.0, a generic instrument developed for adults to assess health and disability, has been adapted for use in children in a number of settings [66,67,68]. The culturally neutral, WHO Indicators of Infant and Young Child Development caregiver report tool, which monitors pre-school children across multiple LMIC settings, has been developed with feasibility testing and piloting across a number of LMIC planned [69].

Second, for children in whom development disability is identified, access to health and rehabilitation interventions to improve functioning and quality of life are limited. Even when available, the uptake is low with numerous barriers to access including cost, transport, physical inaccessibility, lack of appropriately trained healthcare workers, as well as cultural beliefs and stigma around disability [70,71]. Moreover, caregivers are likely to experience high levels of depression and anxiety with limited family and community support [72].

Third, children with developmental disabilities are often excluded from programmes and clinical trials of early child development interventions as it may be difficult to quantify improvement when developmental progress is the primary outcome, further compounding delay and reducing learning opportunities [73].

Fourth, there is a dearth of good quality information from large studies on the spectrum of evolving disabilities in children of all ages post TBM and limited information from LMICs on what interventions are effective for children with disabilities in general, their cost effectiveness and scalability. Collaborative efforts between stakeholders, funders and researchers, will help to improve outcome for children and families dealing with the sequelae of TBM. As TB clinicians and researchers, we must strongly advocate for these initiatives (Table 5).

## 6. Pillar 5: Advocacy and Engagement

Advocacy is key to bringing about changes to policies and practices at institutional, community and individual level [74]. Advocacy for TBM fits into the scope of the meningitis roadmap by working with partners to raise public and political awareness of TBM and its devastating effects in order to improve diagnosis, treatment, prevention and support for affected families [4].

There are existing frameworks for TB control that advocates for TBM can leverage. The End TB strategy envisions the world free of TB, with zero deaths, disease, and its catastrophic consequences [75]. It is well known that TBM has devastating sequelae and contributes to significant morbidity and mortality. The child and adolescent TB roadmap aims to draw attention to the childhood TB epidemic and has placed advocacy and fostering partnerships as one of its key actions points [6]. Within these frameworks, global partnerships and national TB programmes can be utilised to raise the profile of TBM and highlight its significant contribution of TB-related mortality in adults and children. This will ensure that strategic interventions are formulated and planned with the aim of increasing its detection, treatment, and prevention. These interventions should include promotion of operational research to better understand the burden of TBM disease, its outcomes and consequences outside what is routinely monitored in country TB programmes. Partnerships with the academic community, civil society organisations, community, and patient advocacy groups can play a key role in raising the profile of TBM and developing champions that serve as key leaders in advocacy for TBM.

Existing advocacy materials for TB can be included with enhanced messaging, raising awareness of communities about the signs and symptoms of TBM in order to improve early recognition and promote early healthcare-seeking. In addition, healthcare providers, who are fundamental to diagnostic and care pathways, should receive continued education to improve their ability to recognise and diagnose the disease, together with managing the sequelae and other neurological disabilities that often arise from TBM. Furthermore, TBM should be incorporated into advocacy for syndromic neuro-disabilities such as epilepsy and cerebral palsy. Research funders should be targeted to harness resources for research in novel, non-invasive, affordable diagnostics and more effective or shorter treatment regimens.

Community messaging should highlight the importance of BCG vaccination and counter vaccine hesitancy that is increasing worldwide. In addition, advocacy for effective household contact tracing and provision and monitoring of TPT for high-risk groups, including individuals living with HIV, should be promoted and strengthened within national programs with communities recognising their rights to this. This combination of efforts has the potential to prevent severe forms of TB in vulnerable populations and the catastrophic costs associated with TBM (Table 6).

## 7. Conclusions

Both the “Defeating Meningitis by 2030: global roadmap” and “End TB Strategy” are ambitious plans, whose timelines for success are likely to be hampered by the COVID-19 pandemic. Collateral impacts from the pandemic include impact on the resilience of health systems, reporting and surveillance structures, health-seeking behaviours, together with cuts in funding from major donors [4,76]. Nevertheless, the pandemic also brings opportunities and provides an opportune moment to consider children with TBM who have amongst the highest burdens of morbidity and mortality of any of the conditions encompassed by the two plans. Although there are evident constraints and limits to how much the “Defeating meningitis roadmap” can be applied to children with TBM, creative and collaborative working between clinicians, policymakers, public health, and community and advocacy organisations can help bring us closer to defeating paediatric TBM.

## Figures and Tables

**Table 1:** Specific reference to tuberculous meningitis in the main text of recent key World Health Organization policy documents for either tuberculosis or meningitis *.

Document	References to TBM
Defeating meningitis by 2030: a global road map (26 October, 2020 draft) [4]	“[The road map] will complement other global control strategies, such as those addressing sepsis, pneumonia, tuberculosis and HIV.” (p. 3) “Estimated cases and deaths due to tuberculous and cryptococcal meningitis are categorised under tuberculosis, HIV or other infectious diseases and are not included in these figures.” (p. 4) “Although the focus of this road map is not on other important causes of meningitis, such as tuberculosis, Cryptococcus, enteric bacteria and viruses such as enterovirus, several goals aimed at reducing the burden of disease are applicable to all causes of meningitis.” (p. 6)
Global Tuberculosis Report 2020 [5]	Bacille Calmette-Guerin “[BCG] provides moderate protection against severe forms of TB (TB meningitis and miliary TB) in infants and young children.” (p. 125) Mention of “High-dose rifampicin for TB meningitis (ReDEFINe) study” on p. 183 and p. 186.
Roadmap towards ending TB in children and adolescents 2018 [6]	Within “specific age- and disease-related challenges” box (p. 8): “Young children are at increased risk of developing severe forms of TB disease (e.g., disseminated TB, TB meningitis) with increased risk of death (especially children < 2 years).” and “TB is frequently missed as underlying cause or co-morbidity of children presenting with pneumonia, malnutrition or meningitis.” Within “Improving recording and reporting of detected TB cases, TB-related deaths and prevention” (p. 10): “Fatal cases of TB that present as severe pneumonia, HIV, malnutrition or meningitis are attributed to these conditions.” Within “Implement integrated family-and community-centred strategies” (p. 15): “Ensure children and adolescents with other common co-morbidities (e.g., meningitis, malnutrition, pneumonia, chronic lung disease and HIV infection) are routinely evaluated for TB.”

* Documents primarily related to meningitis were searched for “TB” or “tuberculosis” whilst documents primarily related to tuberculosis were searched for “meningitis.” p denotes page.

**Table 2:** Selected strategic goals reproduced from Pillar 1 of the “defeating meningitis” roadmap and suggested paediatric tuberculous meningitis (TBM)-related activities [4].

Adapted Strategic Goals from “Defeating Meningitis” Road Map	Suggested Key Activities Adapted to Paediatric TBM
Strategic Goal 1: achieve and maintain high coverage of licensed WHO vaccines with equal access in all countries and introduce these vaccines in countries that have not yet introduced them in line with WHO recommendations.	Implement locally appropriate tailored immunization strategies to achieve and maintain high BCG vaccination coverage, reinforcing and complementing existing immunization strategies, including those targeting special risk groups. Ensure effective linkages and synergies between WHO, UNICEF, Gavi, the Vaccine Alliance, and other global or regional initiatives aiming to reduce price and increase sustainable access to both BCG and future novel licensed vaccines for LMICs.
Strategic Goal 2: introduce effective and affordable new WHO prequalified vaccines.	Support development, licensure, WHO prequalification and introduction of effective, affordable, and safe new TB vaccines. Improve support to vaccine manufacturers in their efforts to ensure diversification of sufficient quality-assured vaccine production capacity in more countries, including LMICs.
Strategic Goal 3: develop evidence-based policy on vaccination strategies that result in optimal individual protection and, where possible.	Enable and promote the sharing of knowledge between countries (for example, on accurate cost-effectiveness models) to support national policy decisions, particularly in low-incidence settings. Assess the overall vaccine impact, duration of protection, and indirect effects induced with BCG (and evaluation of strain-specific differences) and novel TB vaccines to inform vaccination strategies to maximise long-lasting immunity in populations and to prevent/control vaccine-preventable TB among at-risk individuals. Establish immune correlates of protection against TB Quantify the potential benefits of BCG and novel vaccines on reducing multidrug-resistant TB.

LMICs = low and middle-income countries.

**Table 3:** Selected relevant strategic goals reproduced from Pillar 2 the “defeating meningitis” roadmap and suggested paediatric TBM-related activities [4].

Adapted Strategic Goals from “Defeating Meningitis” Road Map	Suggested Key Activities Adapted to Paediatric TBM
Goal 6: improve diagnosis of TBM at all levels of care.	Improving time to diagnosis. CSF rapid diagnostics tests performed in TBM. Increased availability and usage of Xpert MTB/RIF Ultra as the initial test. Further evaluation of performance of TB-LAMP in TBM which can detect DNA in less than 1 h. Further evaluation of mycobacterial antigen tests such as LAM in CSF in TBM. Active research into blood/CSF biomarkers which distinguish TB meningitis from other CNS infections. Non-CSF rapid diagnostic tests. Further evaluation of blood-based biomarkers (RNA, protein, metabolite). Further evaluation of urinary LAM in TBM. Improving sampling and laboratory processes to optimise yield. Greater emphasis on optimal CSF volumes for diagnosis and safety of obtaining these volumes. Ongoing training of laboratory staff. Maintenance and upgrading of laboratory equipment. Improving healthcare worker understanding of timely diagnosis, referral, and treatment. Development and adoption of standardised international guidelines on diagnosis, referral, and treatment of TBM particularly in LMICs. Greater emphasis on TBM within national TB programs. Accessible education of front-line healthcare professionals regarding thresholds and indications for lumbar puncture. Qualitative research to understand, from communities and health professionals, the factors contributing to low parental acceptance of lumbar puncture in different settings and evidence-based measures to increase acceptance.
Goal 7: develop and facilitate access to diagnostic assays at all levels of care to increase confirmation of TBM.	Funding mechanisms to facilitate development and uptake of novel rapid diagnostic assays. Partnering with diagnostic companies to evaluate new and rapid diagnostic tests. Partnerships with research grant funding agencies to evaluate diagnostic tests. Mechanisms for validation, production and adoption of diagnostic assays. Development of diagnostic assays to support immediate medical decision making at point-of-care.
Goal 8: develop and implement a context specific policy to identify mothers who have TB disease in pregnancy and post-partum, and for diagnosis of neonatal TB, particularly for low-resource settings.	Develop and implement a context-specific strategy for diagnosis, particularly for low-resource settings. Updating national TB guidelines to include diagnosis of maternal and neonatal TB, identification of infants. at risk of disseminated TB or TBM, and provision of TPT.
Goal 9: provide and implement appropriate, context specific, quality-assured guidelines and tools for treatment and supportive care to reduce the risk of mortality, sequelae, and antimicrobial resistance.	Review evidence on potential benefit of adjunctive therapies for bacterial meningitis (steroids/aspirin/thalidomide) in LMICs. Develop and implement updated evidence-based regionally adapted guidelines and recommended tools on patient treatment and care for all age groups from early diagnosis to early identification, treatment, and care of sequelae, and addressing antimicrobial resistance and integration into existing guidelines. Ensure that recommended and quality-assured antimicrobials and medical supplies needed for supportive care are affordable and accessible at country level including paediatric formulations.

CSF = cerebrospinal fluid, WHO = World Health Organisation, MTB = mycobacterium tuberculosis, Rif = rifampicin resistance, TB = tuberculosis, TBM = TB meningitis, HIV = human immunodeficiency virus, LAM = lipoarabinomannan, LAMP = loop-mediated isothermal amplification, TPP = target product profile, LMIC = low and middle income countries.

**Table 4:** Selected relevant strategic goals reproduced from Pillar 3 the “defeating meningitis” roadmap and suggested paediatric TBM-related activities [4].

Adapted Strategic Goals from “Defeating Meningitis” Road Map	Suggested Key Activities Adapted to Paediatric TBM
Strategic Goal 10: ensure that effective systems for surveillance of meningitis and detection of the main meningitis pathogens are in place.	Improved reporting of different sites of extrapulmonary TB, including TBM. Triangulation of laboratory, public sector healthcare, and private healthcare notifications of TBM diagnoses. Specific reporting of MDR-TB TBM given the accompanying therapeutic challenges. Improved access to global genome partnerships for TB, including TBM.
Strategic Goal 12: develop and conduct surveys and studies to establish the burden of sequelae.	Develop and implement a global strategy and tools for studies and surveys to establish and monitor the burden of TBM sequelae.

**Table 5:** Selected relevant strategic goals reproduced from Pillar 4 the “defeating meningitis” roadmap and suggested paediatric TBM-related activities [4].

Adapted Strategic Goals from “Defeating Meningitis” Road Map	Suggested Key Activities Adapted to Paediatric TBM
Strategic Goal 13: strengthen early recognition and management of sequelae from meningitis in healthcare and community settings.	Conduct research on: (i) socioeconomic impact of sequelae on children, adults and their families/carers; (ii) effectiveness of aftercare/support interventions in reducing impact. Develop and implement best practice guidelines for LMICs on detection, monitoring and management of TBM sequelae after discharge from hospital, at all levels of healthcare and in community settings, for example, schools (including disability sensitization and communication skills). Promote community-based programmes to: (i) Identify sequelae and disabilities, based on standardised instruments (especially for child development and hearing), and refer for assessment and appropriate care. (ii) Provide care, support and aftercare to individuals, families and communities affected by TBM, for example, psychosocial support.
Strategic Goal 14: increase the availability and access to appropriate care and support (i) for people affected by meningitis; (ii) for their families and carers.	Map out existing services and support systems by country for: (i) children and people with disabilities, including those with TBM sequelae, and (ii) for families/carers of people affected by TBM; identify barriers to access, availability, and use, with the involvement of organisations for persons with disabilities and other networks where possible, and undertake a gap analysis to improve service provision. Strengthen partnerships between government and civil society organisations, including organisations for persons with disabilities and other networks, so that people with sequelae or disabilities, their families/carers and those bereaved due to TBM have access to quality and effective services that are in line with international human rights standards and frameworks. Provide relevant, up-to-date information to people and carers affected by TBM about access to services for managing sequelae as well as about the rights of people with disabilities guaranteed under national policies and laws and through global human rights instruments.

**Table 6:** Selected relevant strategic goals reproduced from Pillar 5 the “defeating meningitis” roadmap and suggested paediatric TBM-related activities [4].

Adapted Strategic Goals From “Defeating Meningitis” Road Map	Suggested Key Activities Adapted to Paediatric TBM
Strategic Goal 15: ensure that funders and policymakers at the national, regional, and global levels recognise that the road map to defeat meningitis is prioritised and integrated into country plans at all levels.	Raise awareness of TBM as a health priority. Among funders and policymakers through national and international champions, civil society organizations, advocacy groups and healthcare providers, including the disability sector. Identify and create synergies between key activities on strategy, implementation, and communication with other initiatives at the global, regional, and national levels, especially for the immunisation and disability sectors. Build a business case for investment in vaccines, surveillance, diagnosis, and treatment of meningitis, and for the prevention and management of sequelae, as set out in the road map, that is targeted for use by policymakers, decision-makers, and funders at the global, regional, and national levels including the disability sector. Countries undertake needs assessment on TBM and its impact and create national action plans that address gaps and are aligned to the global road map. Develop communications and engagement strategy and improve global recognition of World Meningitis Day, World TB day and other global health dates (for example—cerebral palsy, disability), adapt messaging to policymakers as well as to the public, and raise funding to promote activities that support the road map.
Strategic Goal 16: ensure awareness, among all populations, of the symptoms, signs, and consequences of meningitis so that they seek appropriate healthcare.	Undertake integrated communication programmes and activities that increase population awareness of the risk, symptoms, signs, and consequences of TBM, and of the recommended health-seeking response, and create community awareness of TB disease and prevention. Study the community understanding of the risk of TBM, and the factors that facilitate or act as barriers to health-seeking behaviours for TBM, and integrate actions into country plans to address the issues identified.
Strategic Goal 17: ensure and raise awareness of communities about the impact of meningitis and available support after meningitis.	Support global and national campaigns on the International Day of Persons with Disabilities to increase and raise awareness of communities about disability, and to address significant attitudinal barriers that lead to stigma and undignified treatment of people with disabilities. Raise awareness of new systems for data collection on sequelae/disabilities and of available support and specialist services. Identify, encourage and support civil society organisations that do or could promote the interests of those affected by TBM, including those with sequelae, and invite involvement in delivering the goals of the road map through their communities, engagement with national and regional authorities and international networks of civil society organizations. Study community understanding of BCG and new TB vaccines, TPT and other preventive strategies.
SG19: maintain high vaccine confidence	Develop risk and communication strategies to address issues of access, acceptance, and generation of demand for BCG and novel TB vaccines. Develop risk and crisis communication plans for BCG and new TB vaccines to address potential inaccurate communication of adverse events.
